# 3-D Ultrastructure of *O. tauri*: Electron Cryotomography of an Entire Eukaryotic Cell

**DOI:** 10.1371/journal.pone.0000749

**Published:** 2007-08-15

**Authors:** Gregory P. Henderson, Lu Gan, Grant J. Jensen

**Affiliations:** Division of Biology, California Institute of Technology, Pasadena, California, United States of America; University of Cambridge, United Kingdom

## Abstract

The hallmark of eukaryotic cells is their segregation of key biological functions into discrete, membrane-bound organelles. Creating accurate models of their ultrastructural complexity has been difficult in part because of the limited resolution of light microscopy and the artifact-prone nature of conventional electron microscopy. Here we explored the potential of the emerging technology electron cryotomography to produce three-dimensional images of an entire eukaryotic cell in a near-native state. *Ostreococcus tauri* was chosen as the specimen because as a unicellular picoplankton with just one copy of each organelle, it is the smallest known eukaryote and was therefore likely to yield the highest resolution images. Whole cells were imaged at various stages of the cell cycle, yielding 3-D reconstructions of complete chloroplasts, mitochondria, endoplasmic reticula, Golgi bodies, peroxisomes, microtubules, and putative ribosome distributions *in-situ*. Surprisingly, the nucleus was seen to open long before mitosis, and while one microtubule (or two in some predivisional cells) was consistently present, no mitotic spindle was ever observed, prompting speculation that a single microtubule might be sufficient to segregate multiple chromosomes.

## Introduction

The history of cell biology has been punctuated by major advances in imaging technologies. Following the invention of the electron microscope in the early 1930s, what we would now call the “conventional” specimen preparation methods of chemical fixation, dehydration, plastic-embedding, sectioning, and staining were developed to allow the visualization of biological material. While producing some artifacts, these methods have been profoundly successful, filling our textbooks with information about the structure and positions of cell walls, internal membranes, cytoskeletal filaments, and even large cytoplasmic particles like ribosomes. High-pressure freezing/freeze substitution fixation techniques have since improved specimen preservation [1].

Recent technological developments have created the opportunity to realize two major improvements: imaging cells in three dimensions (3-D) and in nearly-native states. Cells can be imaged in 3-D through tomography [2], a technique wherein specimens are imaged iteratively while being incrementally tilted around one or two axes. Full 3-D reconstructions of the sample can then be calculated. Computationally stacking such 3-D reconstructions of serial (thick) sections has made it possible to reconstruct even large regions of fixed cells [3]. With conventional sample preparation methods, however, image contrast arises from heavy metal salts surrounding cross-linked molecules held in place by a resin support. It has now also become possible to image non-chemically-fixed cells in a more nearly native state through plunge-freezing. Plunge-freezing preserves cells in a life-like, “frozen-hydrated” state free of stains and with minimal artifacts [4]. Electron cryotomography (ECT) combines these two advances to produce 3-D reconstructions of nearly-native biological material to “molecular” resolution [5].

Because of multiple scattering, however, images of samples thicker than about 500 nm are difficult to interpret. Thus previous ECT work has focused on purified macromolecules, viruses, small prokaryotic cells [6], purified organelles [7], or cell peripheries [8], [9], but the potential insight that might come from examining whole eukaryotic cells by ECT has not yet been realized because they are too thick. To this end methods are being developed to either cryosection frozen-hydrated tissues [10] or focused-ion-beam mill thin slabs [11] suitable for tomography. Here we have taken an alternative approach by identifying and imaging the smallest known eukaryotic cell, *Ostreococcus tauri*.

*O. tauri* is a unicellular green alga (a “picoplankton”) with only a single mitochondrion, chloroplast, and Golgi body [12]. It has been reported to be less than a micron in diameter, and because of its small size and presumed simplicity, it has been put forward as a model eukaryotic organism [13]. Nevertheless when *O. tauri*’s 12.6 megabase nuclear genome was sequenced [13], it was found to have more genes (∼8,200) than *Saccharomyces cerevisiae* (∼6,600). These genes are distributed on 20 linear chromosomes. The Prasinophyceae lineage to which *O. tauri* belongs represents an early branch from the green lineage that includes the land plants [14], [15]. Unlike other model plants *O. tauri* has only one copy of each class of cyclin-dependent kinases and cyclins [16], making it a powerful system to study the cell cycle without concerns of genetic redundancy. *O. tauri*’s cell cycle can be partially synchronized by growing the cells in a twelve hour light-and-dark cycle and further synchronization can be achieved pharmacologically [17]. Because of its small size, it may be the only eukaryotic cell known that can be effectively imaged intact throughout its entire life cycle by ECT.

Here we present ECT reconstructions of intact *O. tauri* cells frozen at different stages throughout the cell cycle. In addition to providing new insights into the ultrastructure of chloroplasts, mitochondria, endoplasmic reticula (ER), Golgi bodies, peroxisomes, microtubules, and the distribution of large macromolecular complexes, *O. tauri* presented a number of surprises including a nuclear envelope (NE) that was open throughout most of the cell cycle, an unusually shaped ER, and a rough ER that was confined to the outer nuclear membrane. Perhaps most intriguingly, only one or two microtubules were ever observed in individual cells, suggesting that single microtubules might be sufficient to segregate multiple chromosomes. The Results and Discussion are presented one after another together for each topic below to improve the flow of the text, but a Conclusion has been added to place the whole work in context.

## Results and Discussion

### Cell Growth and Synchronization

Cells were obtained from a strain of *O. tauri* isolated from the Thau lagoon (France) [18]. *O. tauri*’s growth was synchronized using a twelve-hour-light, twelve-hour-dark cycle that resulted in an average of one cell division per day. A previous study found that under these growth conditions, cells at the dark-to-light transition were in the mid-G_1_ phase of the cell cycle. Cells at the light-to-dark transition were found in various stages of the cell cycle including approximately 50% in early G_1_, 10% in late G_1_, 25% in S, and 15% in G_2_/M [17].

### Sample Preservation Strategies

**Figure 1:**
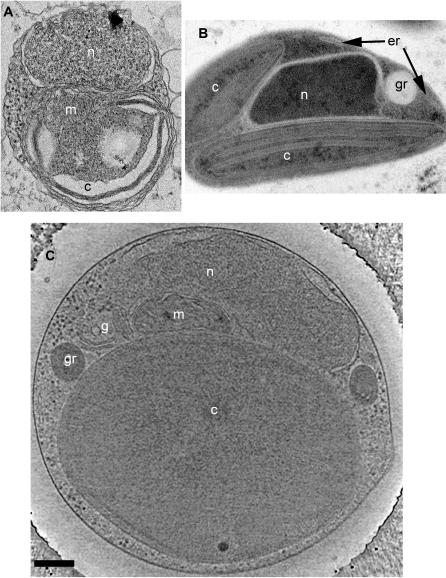
Preservation of *O. tauri*. (A) Projection image through a conventionally fixed, embedded, thin-sectioned cell. (B) Projection image through a high-pressure frozen, low-temperature embedded, thin-sectioned cell. (C) 28.8-nm thick slice through the 3-D electron cryotomographic reconstruction of an intact, frozen-hydrated cell. The letters identify nuclei (n), chloroplasts (c), mitochondria (m), Golgi bodies (g), the Endoplasmic Reticulum (er), and granules (gr). Scale bar = 200 nm.

Cells from liquid cultures were prepared for EM using three different preparation methods: conventional fixation and embedding, high-pressure freezing/freeze-substitution fixation, and plunge-freezing (Figure 1). Conventional EM fixation revealed most membranes, but the membrane structures were badly distorted and any further details were difficult to resolve and/or poorly reproducible. High-pressure frozen specimens had markedly improved membrane preservation, especially in the chloroplast, but macromolecular complexes were not resolved. Further, because of stain and the generally high-density of the plastic-embedded samples, sections thick enough to contain entire cells could not be imaged. Thus reconstructing an entire cell would have required multiple serial sections. Plunge-frozen cells were imageable intact and exhibited good ultrastructural preservation including readily resolvable ribosome-like particles, microtubules, and smooth cellular membranes. Moreover, plunge-freezing was both more consistently successful and significantly faster than high-pressure freezing, allowing a large number of whole cells to be imaged and a more comprehensive survey of *O. tauri* ultrastructure to be obtained. The plunge-frozen cells were, however, at the limit of useful thickness for ECT (500–800 nm), so data could not be collected at tilt angles greater than ∼55–60° and the resolution of the reconstructions was correspondingly anisotropic.

### Data Recorded

**Figure 2:**
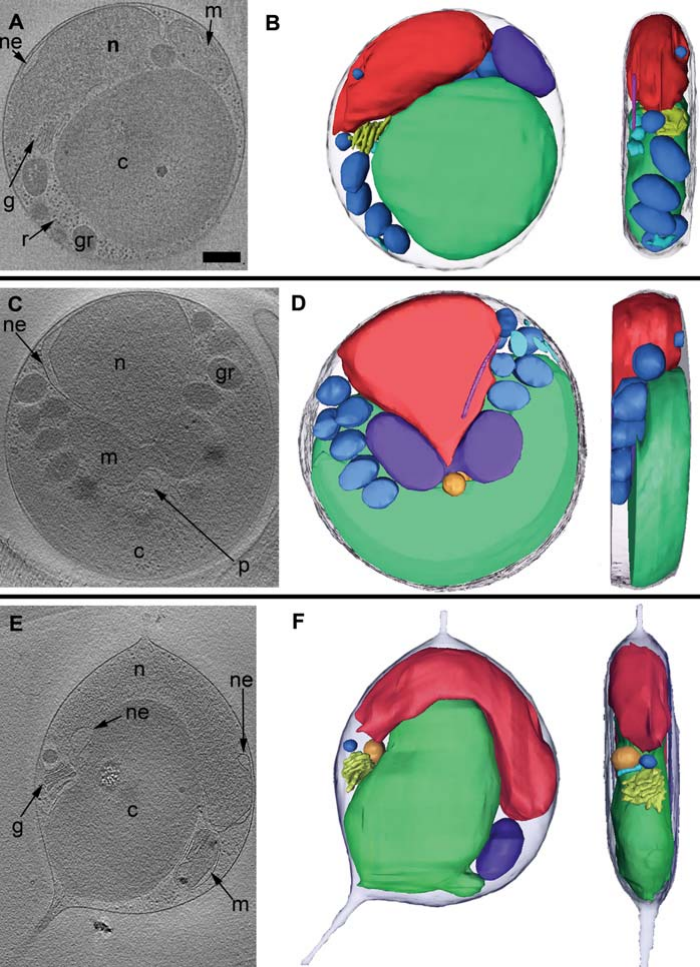
Cross-sections and 3-D segmentations of *O. tauri* cells. Each row shows a single slice (A 21.6-nm; B 36-nm; C 9.6-nm) through the middle (left) and a manually segmented model (two perpendicular views, right) of one particular cell: (A, B) a non-dividing cell harvested at the dark-to-light transition; (C, D) a dividing cell harvested at the light-to-dark transition; (E, F) an unusual cell with a different internal organization and two membrane-bound cell extensions. Only the central portion of the cell in (F) is shown due to the limited ability to completely segment thicker cells. Here and below the letters and colors identify nuclei (n, red), nuclear envelope (ne), chloroplasts (c, green), mitochondria (m, dark purple), Golgi bodies (g, yellow), peroxisomes (p, orange), granules (gr, dark blue), inner membranes including ER (er, light blue), microtubules (light purple), and ribosome-like particles (r). Because the dividing cell shown in panels C and D was especially thick, only its middle region was segmentable. Scale bar 250 nm.

Cultures at both the dark-to-light and light-to-dark transitions were plunge-frozen. Samples were first surveyed in the EM to investigate the cell sizes and morphologies present. Cells were almost always round, and were seen to vary in diameter continuously from 1 to 2 µ on every grid, but cells were generally larger at the light-to-dark transition. In many of the cells frozen at the light-to-dark transition, it was possible to see in the surveys that the organelles were dividing. Full tilt-series of 52 cells were then recorded. Most of these were chosen because they were especially thin, which results in higher resolution reconstructions, or because they had visibly dividing organelles, but some were chosen almost randomly to broaden coverage of the cell cycle. The reconstructed cells were classified into two groups, “non-dividing” and “predivisional,” based on whether or not their organelles were visibly dividing (Figure 2 and in particular see Video S1, which presents a complete 3-D tour of one representative cell and all its organelles). Of the 23 reconstructed cells taken from the dark-to-light transition, all but one were non-dividing, consistent with their being in G_1_ as predicted by Farinas *et al*, 2006. Of the 29 reconstructed cells taken from the light-to-dark transition, 4 were non-dividing and 25 were predivisional.

### Cell shape and content

**Figure 3:**
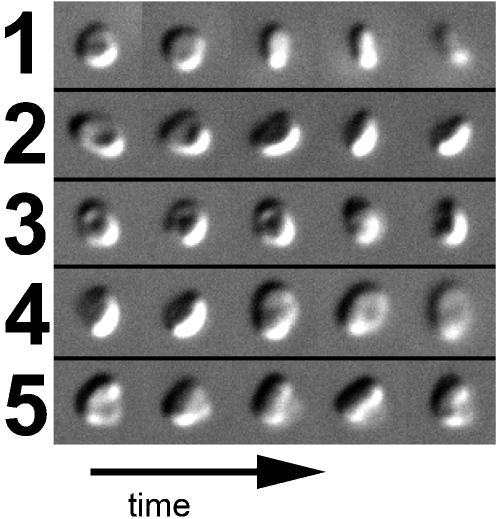
Light microscope images of free-floating *O. tauri.* Sequential DIC images of five representative cells (1–5) tumbling freely in sea water culture medium after six hours growth in light are shown from left to right, demonstrating their disk-like shape.

While the limited tilt range decreased the resolution perpendicular to the grid, it was nevertheless clear that the frozen-hydrated cells were not spherical. Instead they were rather flattened, ranging from 1.1 to 2.1 µm in diameter and 0.5 to 0.8 µm in thickness. The more flattened surfaces are referred to herein as the “top” and “bottom” of the cells, depending on their orientation on the grid, but there were no consistent structural features that indicated any functional distinction. The diameters and flatness observed here were consistent with earlier reports [12], but to check whether surface tension flattened the cells beyond their normal state, cell shapes were also investigated by phase and differential interference contrast light microscopy of free-floating live cells. Free-floating cells also had flat profiles, but exhibited more complex morphologies with bends and depressions as if they were quite flexible (Figure 3). Thus while the flattened morphology is natural, suspending the cells in thin fluid across circular holes on an EM grid likely promoted a more round and simple shape.

All cells imaged by ECT contained a single mitochondrion, Golgi body, and chloroplast. Each cell also contained a nucleus with one to three discernable nuclear pore complexes (NPCs), a smooth ER, and a variety of vesicles that could not be identified. The non-dividing cells had a single peroxisome and microtubule, but some predivisional cells had two copies of each. The rough ER was limited only to the outer NE. One unusual cell had an oval shape with two terminal membrane-bound cell extensions (Figure 2E–F). This cell was thinner than the others, had the long axis of the chloroplast turned ninety degrees relative to the long axis of the nucleus, and had giant holes in the NE.

### Cellular Organization

**Figure 4:**
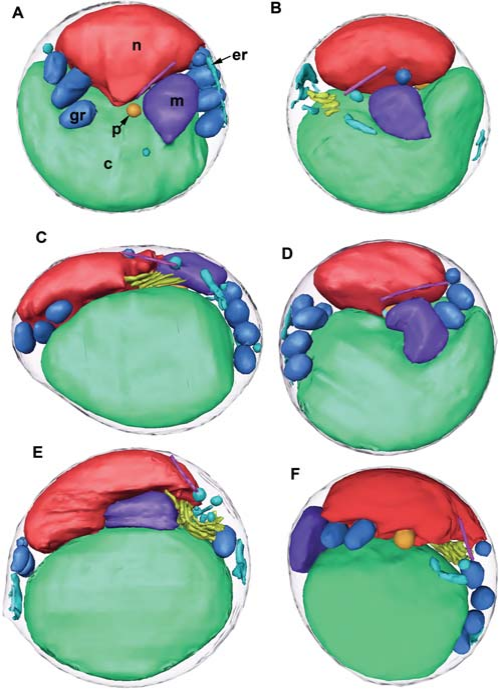
3-D segmentations of six cells imaged at the dark-to-light transition (mid G_1_). The cell in panel F is the same as in Figure 1A–B and 12. For scale, the diameter of the cell in panel D is approximately 1750 nm in diameter.

The organization of the non-dividing cells was remarkably consistent. The largest organelles – the chloroplast and nucleus – resided in opposing semi-circular halves of the cell. The other organelles (mitochondrion, peroxisome, ER, Golgi body, vesicles) rested between them (Figure 4). The nucleus and chloroplast pressed so closely to the plasma membrane that macromolecular complexes like ribosomes were sterically excluded. While the mitochondrion did not appear to have a fixed location and the chloroplast rested opposite the nucleus, the nuclear envelope (NE) appeared to organize the rest of the organelles. The outer nuclear membrane was contiguous with sheets of ER that were found to run parallel to the plasma membrane and around co-localized granules. The Golgi body was always found close to the nucleus. A single microtubule could be found in contact with the NE, near the top or bottom of the cell. The peroxisome, a large single-membrane vesicle known to be present in all eukaryotic cells, occupied the central region of the cell between the nucleus and the chloroplast (discussed later).

Six of the best reconstructed cells were used to estimate the volumes and surface areas of key organelles. These six (shown in Figure 4) were chosen because they were among the thinnest cells and because they were imaged along two orthogonal axes, a practice that minimizes the effects of the missing wedge of data in ECT [19]. All six came from the dark-to-light transition. These presumably underwent cell division at the beginning of the 12-hour dark period, entered G_1_, and then grew minimally due to lack of light. The average cell volume and surface area was 0.91±0.07 µm^3^ and 8.3 µm^2^, respectively. The chloroplasts, nuclei, and mitochondria were the largest three organelles, occupying 47±3, 17±2, and 2.7±0.4% of the cell volume, respectively. The cytoplasm around the organelles occupied another 29±2% of the cell and granules and other vesicles occupied the remainder of the volume. In these thinnest of cells, the distribution of organellar volumes was remarkably consistent.

**Figure 5:**
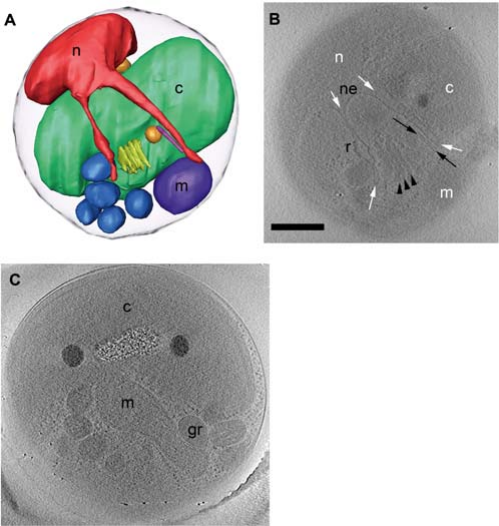
Cells with dividing organelles. (A) Segmentation showing a portion of a NE streaming over the top of a chloroplast. Here the chloroplast is separated from the plasma membrane by the mitochondrion and several granules. (B) 36-nm thick slice near the top of the reconstruction of the same cell. The NE (white arrows) stretches towards the Golgi body (black arrowheads) and microtubule (black arrows). (C) 24-nm slice through a different cell, showing an elongated mitochondrion near the center of the cell and duplicating chloroplast granules. Scale bar = 150 nm.

**Figure 6:**
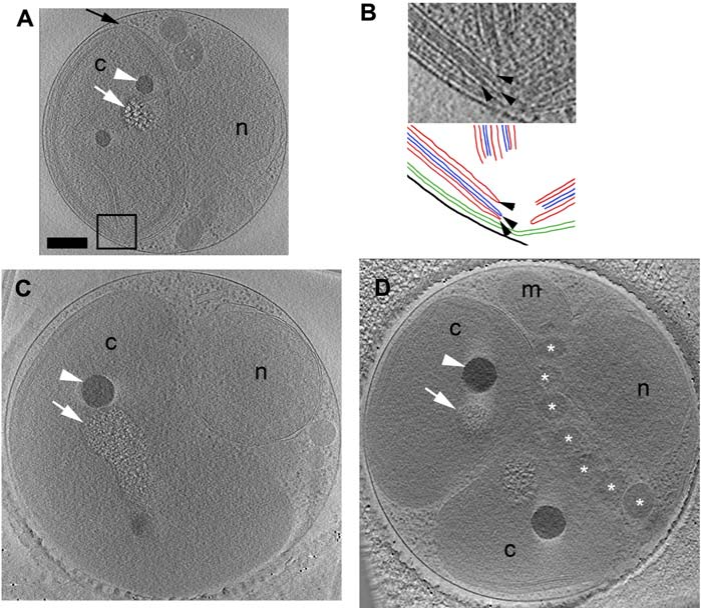
Chloroplast. (A) 7.2-nm thick slice through a non-dividing cell. The starch granule (white arrow) has suffered damage from the electron beam. Besides it are two dark granules (white arrowhead). (B) Above: Enlarged view of the boxed area in panel A. The three thylakoid membranes (black arrowhead) can be seen forming a stack. Below: Schematic of above. Cell membrane (black), chloroplast membranes (green), outer thylakoid membrane (red), inner thylakoid membranes (blue). (C) 24-nm slice through an early predivisional cell, where the chloroplast is kidney-shaped rather than oval and the starch granule is elongated. (D) 36-nm thick slice through a late predivisional cell, where the chloroplast is deeply constricted and one dark and one starch granule is found in each side. Here the cytoplasmic granules (*) are arranged in a V-shape pointing to the division plane. Scale bar 250 nm.

The predivisional class of cells was distinguished by four ultrastructural differences. (1) The most obvious difference was the partial division of the chloroplast along a line approximately perpendicular to the boundary between the nucleus and chloroplast, setting up an apparent roughly two-fold-symmetric division plane (Figure 2C–D, 6C, 6D, and 7C). In most of the predivisional cells (62%), there was a space between the chloroplast and the plasma membrane filled with either ribosome-like complexes or the mitochondria (Figure 5A), as if the forces positioning the chloroplast immediately next to the membrane in G_1_ were weakened. (2) The nucleus was no longer oval. In some cases the nucleus was wedge-shaped, protruding into the middle of the dividing chloroplast (Figure 2C–D). In twelve other cases (46%) the NE formed extensions that streamed above/below the chloroplast, sometimes accompanied by the Golgi body or microtubule (Figure 5A–B). (3) Whereas in G_1_ the mitochondrion appeared to be randomly positioned, in dividing cells at least part of it consistently crossed the apparent division plane. In some cases it seemed to be straddling the division plane (Figure 2C–D), in others it was elongated as if at least one edge were being drawn to the division plane (Figure 5C). (4) The oval storage granules lined up in a symmetric “V” shape across the division plane pointing towards the chloroplast with the tip of the “V” located at the cell’s center (Figure 6D).

### Chloroplasts

The chloroplast was the largest organelle and since it was also one of the most dense, its internal structure was the most difficult to resolve. Due to the missing wedge artifact, thylakoid grana that may have been present at the top and bottom of the chloroplast could not be resolved. In the midsection, grana composed of three disks (six membranes) hugged the inner chloroplast membrane and sometimes extended into the middle of the chloroplast as broad sheets without connecting stroma-like thylakoid structures (Figure 6A–B). Because the lumens of the two outer disks (about 7 nm across) were consistently wider than that of the central disk, the grana had a sandwich-like appearance: the “top” and “bottom” membranes of the stack stood apart from the inner four, which were packed so tightly that they were often hard to distinguish. The grana thylakoids terminated at the poles of the long axis of the chloroplast (Figure 6A–B), but the connectivity of the individual membranes and the details of the presumed organizing scaffold were unclear. No connections or vesicles between the grana thylakoids and the inner chloroplast membrane were seen.

The chloroplasts contained two distinct classes of granules. Each chloroplast had multiple dark and very nearly spherical granules (Figure 6, white arrowheads) and a single larger granule that manifested electron beam damage artifacts (bubbling) much earlier than the rest of the cell (Figure 6, white arrows). Radiation-sensitive granules have also been reported in the gram-negative bacterium *Caulobacter crescentus* and were found to be carbon-rich [20]. The “bubbled” granule was seen stretched across the division plane in dividing chloroplasts, as expected for the starch granule, which was previously shown to exist as one copy that grew and divided [21]. The onset of bubbling in the granule occurred at ∼100 electrons/Å^2^, since in the cases of those cells where dual-axis tilt-series were recorded, reconstructions obtained from just the first half of the data (the first tilt ∼80 electrons/Å^2^) did not show bubbling. (The higher total doses were used, however, because they led to reconstructions with more interpretable detail, indicating that the perturbations to the rest of the cellular ultrastructure caused by bubbling in the presumed starch granule were minor in comparison to the advantages of higher dose.) The chloroplasts divided along the apparent cellular division plane (Figure 6C–D, 7C). As the thylakoids terminated at poles (Figure 6B) away from the division plane, the division would result in one old pole and one new pole for each half of the chloroplast. Each half of dividing chloroplasts contained a portion of the presumed starch granule and at least one dark granule. No completely divided chloroplasts were observed, suggesting that cell division quickly followed the termination of chloroplast division.

**Figure 7:**
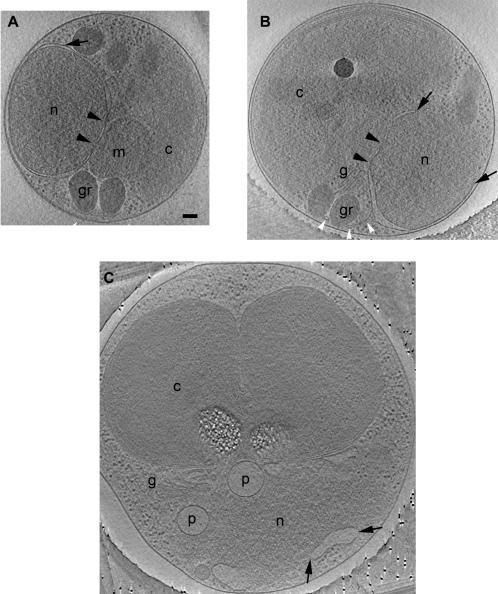
Nuclear envelope. (A) 41-nm thick slice through a cell harvested at the light-to-dark transition with a completely closed NE (black arrow). The cell’s small size and non-dividing organelles suggest the cell could have recently divided. Two NPCs (black arrowheads) are present. Close ups of the NPCs are shown at Figure 8. (B) 31-nm thick slice through a cell harvested at the dark-to-light transition. The NE only covers about three-fourths of the nucleus in this section (black arrows mark the tips). Again two NPCs (black arrowheads) are present, and at the bottom of the nucleus the ER branches off the NE (white arrowheads). (C) 19.2-nm thick slice through a large, late predivisional cell harvested at the light-to-dark transition exhibiting an almost completely open nucleus with only small patches of NE (black arrows). Scale bar 100 nm.

In agreement with the likely evolutionary relationship between chloroplasts and cyanobacteria, the ultrastructure of the *O. tauri* chloroplasts was reminiscent of cyanobacteria. Both thylakoid systems are comprised almost exclusively of grana [22], [23], positioned here near the inner chloroplast membrane and near the analogous plasma membrane in cyanobacteria. The chloroplasts also appeared to replicate like cyanobacteria, since their thylakoids were present before division and must have been pinched off at the nascent poles. In contrast, higher plant chloroplasts mature from progenitor proplastids that lack differentiated internal membranes.

Traditional models depict the lumens of thylakoid disks as discreet from the lumen between the chloroplast's inner and outer membranes [24]. A recent tomogram of a higher-plant chloroplast supports this traditional “detached” thylakoids model [25]. Van de Meene et al. showed a cyanobacteria’s thylakoid contacting the inner plasma membrane, however, but the nature of the connection could not be determined [22]. Here *O. tauri* thylakoids appeared detached, but unfortunately the complete chloroplast could not be clearly observed, transient events might not have been captured, and the details of the presumed organizing scaffolding could not be resolved. No transport mechanisms for bringing required lipids to a growing thylakoid, such as vesicles between the inner membrane and thylakoid stacks, were detected.

### Nuclei

Higher eukaryotes undergo open mitosis, resulting in the partial or complete breakdown of the NE. The onset of mitosis is marked by NE breakdown, NPC disassembly, and mitotic spindle formation. The NE reforms on the chromosomes during metaphase and completely closes after telophase [26]. In contrast, model lower eukaryotes like yeast undergo closed mitosis, in which the NE remains intact. Our reconstructions reveal that *O. tauri*’s NEs are almost always open, and other mitotic events differ substantially from traditional models.

Throughout the cell cycle the NE had gaps hundreds of nanometers in diameter (Figure 7B, arrows). In fact only one cell was observed with an apparently closed NE (Figure 7A). This small cell was taken from a culture at the light-to-dark transition, and its non-dividing organelles implied that it must have just divided and was in early G_1_. This cell could have had gaps in the top and bottom of the NE, but we could not verify this due to the effects of the missing wedge. Other cells imaged that were also presumably in early G_1_ were found to have an open NE. The openings in the NE consistently faced the cytoplasmic space between the nucleus and the chloroplast; the regions facing the plasma membrane were generally closed. In certain rare cases, the nucleus was almost completely unbounded by NE (Figure 7C and 2E). In these cells large fragments of NE were seen along the borders of a recognizably dense nucleoplasm, implying that the nucleoplasm is gel-like and able to retain its unique composition.

The holes in the NE are unlikely to be an artifact of sample preservation; four lines of evidence support the authenticity of these holes. First, no other cell membranes showed similar gaps. Second, nuclei have been previously isolated from cells and imaged by ECT, without disruption of the membrane [27]. Third, the locations of the holes seen here were consistent, which suggests they form in a regulated manner. Finally, some cells simply did have not enough NE to cover the entire nucleus (Figure 2E), arguing against an acute change.

Near the plasma membrane, the outer and inner NE membranes were too close to be resolved. Elsewhere, these membranes were spaced between 18 and 100 nm apart. NPCs were present (discussed below). As noted previous, in 42% of the predivisional cells, protrusions of the nucleus streamed over the top of the chloroplast (Figure 5A–B). No internal structures (like heterochromatin, nuclear lamina, or condensed chromosomes) were recognized.

Many higher eukaryotes undergo NE breakdown during mitosis, so the fact that a cell can have a NE with large gaps is reasonable. Nevertheless *O. tauri*’s NE appeared to be open during most of the cell cycle (from the beginning to the end of the light cycle). This is significant because presumably, the nucleus must be closed in order to establish the Ran nucleo-cytoplasmic gradient, which shuttles cargo in and out of the nucleus via the NPCs. The NE had such large gaps that the role of the NPCs is unclear, although they remained embedded in the NE fragments. Perhaps certain molecules like mRNAs require regulated transport and so are specifically targeted to the NPC. Despite being open, many of the nuclei must have been transcriptionally and translationally active since the cells were in the exponential growth phase and ribosomes consistently decorated parts of the NE.

Previously published EM images of *O. tauri* do not suggest the existence of gaps in the NE [17]. With conventional EM, however, in some cases membranes can be severely distorted and each image generally shows just a thin section through the nucleus (Figure 1). Because ECT offers 3-D views of near-native structures, the gaps were quite clear.

### Nuclear Pore Complexes

**Figure 8:**
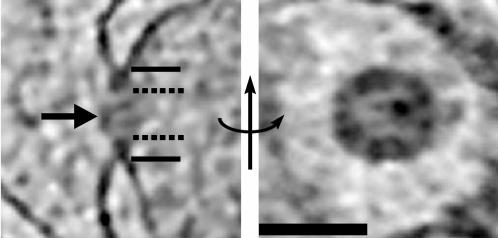
Nuclear pore complex. 3.6-nm thick slices through the best-resolved NPC from the “side” (perpendicular to the NE, left) and “top” (in plane of NE, right). The approximate center, inner, and outer diameters are marked by the arrow, dashed, and solid lines, respectively. The region around the NPC in the right panel is low density (whiter) because it is inside the NE lumen; the dark crescent near the edge of the panel shows where the plane of the slice cuts through the NE. Scale bar = 100 nm.

NPCs within the NE were identified as ∼80 nm diameter rings of high electron density bordered by a region of the NE where the outer and inner membranes merged (Figure 8). Each cell had one to three visible NPCs, but there may have been additional NPCs within the top or bottom faces of the nuclei that were not recognized because of the missing wedge. As mentioned earlier, the nuclei were immediately adjacent to the plasma membrane, but the NPCs were never found facing that membrane. Instead, the NPCs were found in regions of the NE exposed to the cell's interior, but the mechanisms localizing the NPCs remain unclear. In the four best resolved NPCs, the average “outer” diameter of the rings (where the outer and inner bilayers of the NE could no longer be distinguished, Figure 8, solid lines) was 81±4 nm. The average “inner” diameter of the high density ring (Figure 8, dashed lines) was 48±8 nm; the greater variance being due perhaps to the uncertainty in locating the inner boundary. Other internal densities were seen which may represent cargo, “transporters,” or luminal spoke rings [28].

While these may be the first measurements of NPCs *in-vivo*, or of plant or algal NPCs in any state, the *O. tauri* NPCs observed here were smaller (81 nm) than those from *Dictyostelium* (125 nm), *Xenopus* (100 nm), or *Saccharomyces* (100 nm) [27], [29], [30]. Unfortunately the resolution was insufficient to detect the symmetry, as was done for isolated *Dictyostelium* nuclei [27], so it is unclear whether the smaller size results from smaller or fewer constituent proteins. Compared to the *Dictyostelium* NE (the only other intact nucleus imaged by cryotomography), which has such a high concentration of NPCs (∼45/µm^2^) that they exhibit local hexagonal packing [27], [30], *O. tauri* NE had a much lower NPC concentration (∼1/µm^2^).

### Mitochondria

**Figure 9:**
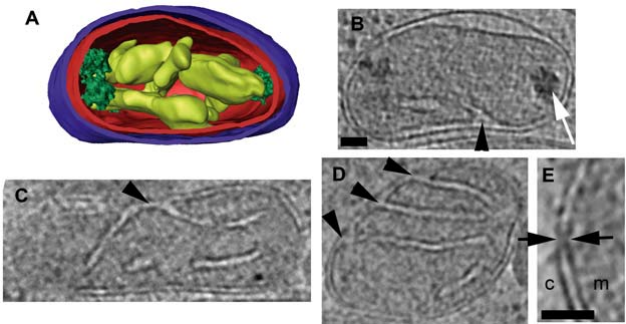
Mitochondrion. (A) 3-D segmentation of the mitochondrion from the cell shown in Figure 1E–F. Here, the membranes are colored purple (outer membrane), red (inner membrane), and yellow (cristae). Dense granules within the mitochondrial matrix are shown in green. (B–D) 15-, 55-, and 29-nm thick slices though three different mitochondria, showing crista junctions (arrowheads) and a dense granule (white arrow). Panel B is a slice through the cell shown in panel A. Scale bar 50 nm (for panels B–D). (E) 4.8-nm thick slice through a junction or channel (black arrows) connecting the outer and inner membranes. Here c-cytoplasm, m-mitochondrion. Scale bar 50 nm.

Each cell had a single mitochondrion. The mitochondria were generally oval (Figure 9), but were frequently deformed when situated adjacent to other organelles. In some dividing cells the mitochondria were elongated (Figure 5C) or constricted in their center (Figure 2C–D). The inner and outer membranes were smooth rather than corrugated as sometimes observed by traditional EM [31]. While the spacing between the inner and outer membrane was generally ∼12 nm (Figure 9C–D), it was sometimes significantly greater (top of Figure 9B), or so small that the inner and outer membranes appeared to touch (Figure 9E). The mitochondria positioned near the thinner edges of cells were the best resolved and exhibited both lamellar and tubular cristae, typically with one or two junctions with the inner membrane (arrowheads in Figure 9B–D). These crista junctions were circular or slot-shaped and ranged in size from 15–55 nm (Video S1). The spacing between the two membranes of the cristae was also 15 nm or greater. In some mitochondria (40%), multiple (4–10) dark granules with complex shapes were observed in the matrix (Figure 9A–B).

Electron tomographic reconstructions of mitochondria have already discredited the baffle model of membrane folding, where cristae were proposed to fold in much like the bellows of an accordion. Instead, both isolated and *in-situ* mitochondria have been shown to have tubular and laminar cristae that connect to the inner membrane by circular or slot-shaped crista junctions [7], [32]. Our observations in *O. tauri* add to this body of literature and highlight the consistency of mitochondrial ultrastructure. Notwithstanding the evolutionary pressures that shrunk *O. tauri* and its mitochondria, the arrangement of its cristae has essentially not changed. This strengthens the idea that the structure of the cristae plays an important role in oxidative phosphorylation, including the possibility that it creates local environments separated by bottlenecks of restricted diffusion [33]–[35].

### Endoplasmic Reticula

**Figure 10:**
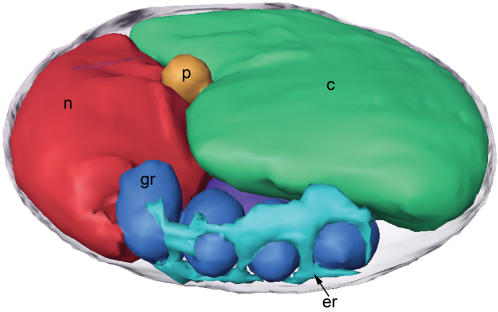
Endoplasmic reticulum. 3-D segmentation of a cell harvested at the dark to light transition. ER (light blue) forms a sheet near the edge of the cell, perforated by four granules (dark blue).

Sheets and tubes of membranes branching off the NE were identified as ER (Figure 7B, white arrowheads). The inter-membrane spacing within each ER sheet ranged from 14 nm to 80 nm. The ER tubes had diameters of ∼14 nm – a value much smaller than the 25–100 nm typically described for smooth ER [36], [37]. Interestingly, some ER sheets were perforated by the dark oval granules (Figure 7B and 10). Other structures looked just like the ER, but connections to the NE were not found. These consisted of sheets that were found adjacent to the plasma membrane and at the four regions that projected extra-cellular filaments, as discussed later.

Current descriptions of ER ultrastructure assert the notions that (1) the lumens of different ER sheets are interconnected with each other and with the NE lumen, and (2) rough ER forms sheets and all other ER (smooth ER) is tubular [36], [37]. *O. tauri*’s ER differed in both these characteristics. While some ER tubes and sheets appeared to be contiguous with the NE, others had no observable connection. Rapid fission and fusion of membranes can make compartments functionally interconnected, like the Golgi body’s many cisternae, but our “snapshots” of flash-frozen cells suggest that some ER compartments are physically isolated at least part of the time. Less likely but still possibly, connections might have existed but were not observed due to the missing wedge artifact or limits in resolution. Surprisingly, while clusters of ribosome-like particles were found on the cytoplasmic face of the outer nuclear membrane, no ribosome-decorated ER (rough ER) could be found. This contradicts earlier speculations that ribosomes are necessary to maintain the ER’s flat shape [37], [38].

### Golgi bodies

Golgi bodies were identified as stacks of flat, membranous cisternae positioned next to the nucleus and usually extending towards the chloroplast. No cell had more than one Golgi body, but in some cells (23%, including both non-dividing and predivisional) no Golgi body was found, probably because it was positioned in such a way that the missing wedge artifacts made it impossible to recognize. In mammalian cells the Golgi body disperses prior to cell division [39]; here the Golgi was frequently observed in predivisional *O. tauri*.

**Figure 11:**
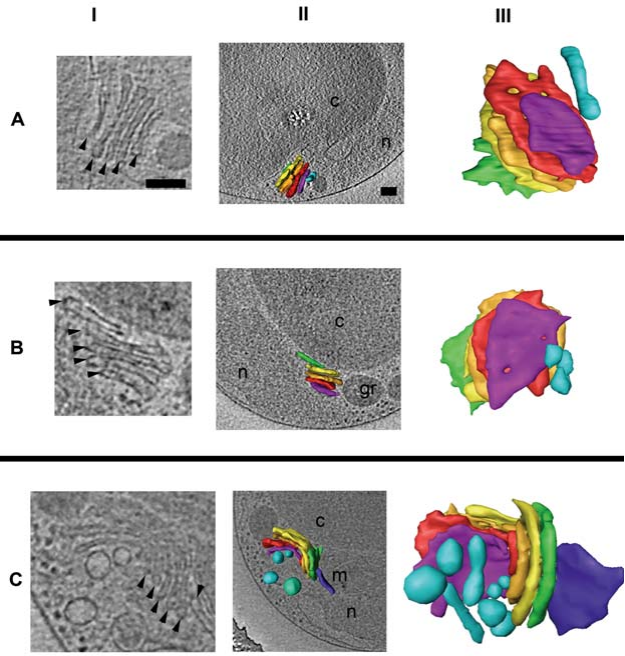
Golgi body. The Golgi bodies from three different cells are shown (rows A–C). In column I, slices through the reconstructions are shown, with cisternae marked by arrowheads. In column II, 3-D segmentations of the Golgi bodies are shown *in situ* within their cellular contexts. Column III shows the isolated 3-D segmentations from a view perpendicular to the one in column II. The five “core” cisternae common to all Golgi bodies seen here are colored purple, red, gold, yellow, and green (*cis* to *trans*). Surrounding vesicles are colored either light or dark blue. Some vesicles in column III do not appear in column II because they are “below” the cellular slice shown. The blue-green vesicle in IIC was removed from IIIC to create an unobstructed view of the Golgi body. Scale bars 100 nm.

In *O. tauri*, the Golgi bodies typically consisted of five uniformly spaced cisternae (Figure 11A–B). Some Golgi bodies exhibited up to seven cisternae, but the extra cisternae were often not consistently spaced or appeared as if they were beginning to leave (Figure 11C, dark blue). The NE was used to determine *cis* to *trans* direction because the resolution was insufficient to resolve the protein coats on the vesicles that are normally used to determine the Golgi polarity. Most cisternae were roughly disk shaped (Figure 11A–B), but more elongated cisternae were also seen (Figure 11C). All were slightly curved. The cisterna nearest the nucleus was consistently the smallest, with a diameter and volume of about 150 nm and 5×10^−8^ pL, respectively. Its concave (cis) surface faced towards the nucleus. The middle three cisternae were larger, measuring 200 nm in diameter and 7.5×10^−8^ pL in volume. The trans-cisterna was 200 nm in diameter and thinner, having a volume similar to the cis-cisterna. None of the cisternae were connected.

The origins and destinations of vesicles in the Golgi are of considerable interest [40]. From the nucleus outwards, occasionally vesicles were seen budding out of the NE towards the cis-cisternae (8% of cells). There were also free-standing vesicles observed between the NE and cis-cisternae. The tubular vesicles in front of the cis-surface (Figure 11, light blue) might have been the fusion of two or more NE-derived vesicles that formed a vesicular tubular cluster. Vesicles were not seen between cisternae, but they were frequently present along the sides of the stacks. The trans-cisterna often abutted the chloroplast, leaving no room for vesicles *trans* to the cisterna. No vesicles were ever found *trans* to the Golgi body, but the trans-cisterna often had an appearance of slipping off the stack (Figure 11A–B, green cisternae), and occasionally had an additional cisterna beyond it (Figure 11C, dark blue cisterna). Vesicles were not observed between the Golgi body and the non-nuclear ER.

These observations are consistent with the maturation model of Golgi body transport, but the lack of *trans* vesicles is surprising. Some of the vesicles along the side of the Golgi body could have originated from the *trans* surface and moved off to the side, sterically deflected by the chloroplast. It may also be possible that instead of having many small *trans* vesicles, here at least Figure 11C suggests that the entire trans-cisternae (dark blue) is one transport vesicle. Many cells rely on microtubules to transport Golgi associated vesicles [41], but as in *Schizosaccharomyces pombe* [3], no microtubules were ever found contacting the Golgi body or its neighboring vesicles. Perhaps other, smaller cytoskeletal filaments were present to guide vesicles but were not resolved in our reconstructions.

### Vesicles

A variety of vesicles were seen. The most numerous type (1–10 or more per cell) were pleomorphic, typically ∼200 nm diameter, and occurred in clusters sometimes configured as a “V” in predivisional cells (Figure 2, 4, 5, 6, 10). Their high internal density prevented us from determining if there was a surrounding membrane. Presumably these are some type of storage granule. Smaller vesicles were also seen whose size and random locations suggest they could have been involved in transport. In addition to these storage and transport vesicles, all the cells (n = 52) contained at least one characteristically low-density, nearly spherical, second type of vesicle surrounded by a single membrane (Figure 4A and F, 10) measuring 166±20 nm in diameter. Some dividing cells had two. Remarkably, these special vesicles were almost always in contact with both the chloroplast and the nucleus (Figure 10), even when they were found far from the main body of the nucleus. In these cases they were found touching long, thin extensions of the NE streaming over the chloroplast (Figure 5A), suggesting connections to both the NE and chloroplast. Because (i) peroxisomes have been described as single-membraned vesicles, (ii) small *O. tauri* cells are likely to need one and only one peroxisome but multiple storage granules, and (iii) *Ostreococcus* genomes contain peroxisomal genes [42], these vesicles were assumed to be peroxisomes. As dividing peroxisomes were never seen, perhaps *O. tauri* only produces peroxisomes *de novo* [43]–[45].

### Microtubules

**Figure 12:**
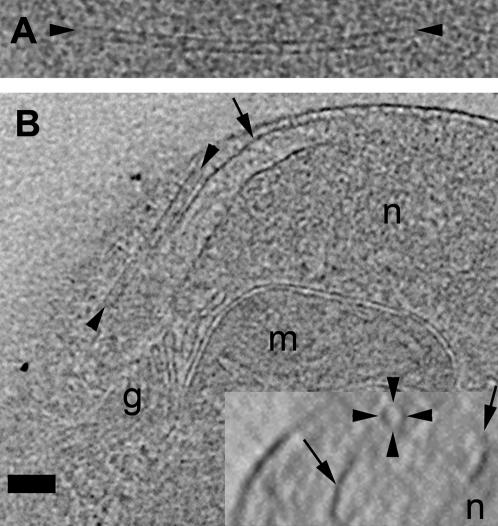
Microtubule. (A) 9.6-nm slice through one particular microtubule (arrowheads) along most of its length, showing its uniform diameter and hollow nature. (B) 16.8-nm slice through a microtubule in its cellular context, showing its open, blunt ends (arrowheads) terminating between the NE (arrows) and plasma membrane. The insert shows a 2-fold enlarged, 38.4-nm thick slice through the microtubule perpendicular to its long axis, emphasizing its tubular shape (arrowheads). Scale bar 100 nm.

Most *O. tauri* cells had a single, hollow, tubular structure 24±1 nm wide and 200–700 nm long assumed here to be a microtubule. The microtubule always rested between the outer nuclear membrane and the plasma membrane against one of the “top” or “bottom” flat faces of the cell (Figure 4, purple rods). Both ends of the microtubule were open and blunt (Figure 12, arrowheads). The microtubules were slightly curved and near one end the shaft made contact with the NE, but the resolution was insufficient to discern whether bridging proteins connected the two (Figure 12B, insert). There was neither an obvious microtubule organizing center nor a flared or capped end to conclusively identify the minus or plus end, respectively. It is possible that the microtubules are organized similarly to those of higher plants, in which the minus end interacts closely with the NE (as discussed later).

Surprisingly, none of the cells exhibited a mitotic spindle even though many stages of division were seen and microtubules were consistently resolved in our reconstructions. Unlike metazoans and yeast, plants lack a structurally distinct microtubule organizing center, but their NEs do organize mitotic spindles that segregate their chromosomes [46]. Three of the reconstructed *O. tauri* cells had two microtubules, and in one of these cells they were partially embedded within the open nucleus (the cell in Figure 7C, though the microtubules are not present in the slice shown), but because *O. tauri* has 20 independent linear chromosomes [13], a canonical mitotic spindle would have been expected to have more than 40 microtubules. While it is possible that in *O. tauri* the spindle appears so transiently that by chance none of the 26 predivisional cells reconstructed here contained one, its absence suggests the intriguing possibility that some evolutionary pressure (like size minimization) has caused *O. tauri* to adapt a novel mechanism for chromosome segregation. Perhaps the twenty pairs of chromosomes are segregated one at a time by the two mitotic microtubules. Alternatively, the chromosomes may be physically linked during mitosis and be co-segregated.

### Ribosome-like complexes

**Figure 13:**
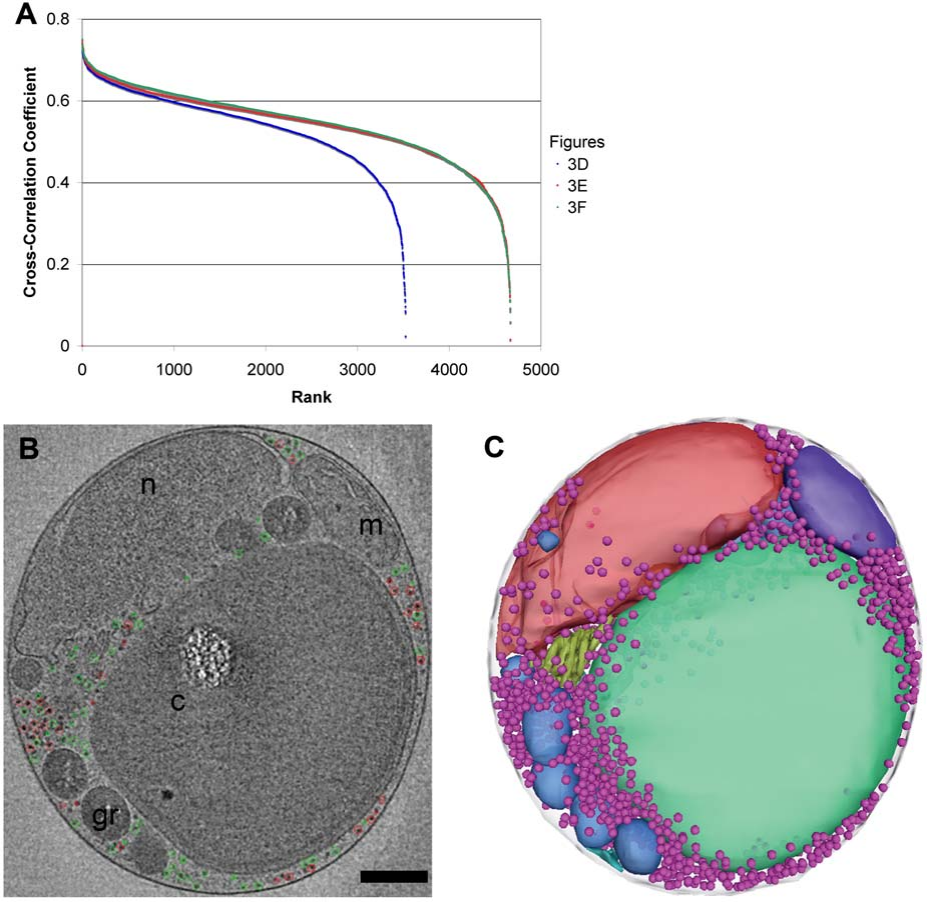
Ribosome-like complexes. (A) Three cells (shown in panels D–F of Figure 3) were searched for ribosome-like particles, and the resulting local-normalized cross-correlation coefficients are plotted from best to worst from left to right. There were fewer total positions ranked in cell “3D” because its cytoplasm was smaller. (B) 24-nm thick slice through a cell with the 500 (red) or 2000 (green) most ribosome-resembling objects in the cytoplasm circled, showing that these are reasonable under- and over-estimates of the total number. (C) 3-D positions of the 1250 most ribosome-resembling objects in the cytoplasm (light purple spheres), showing their close and even distribution. Scale bar 250 nm.

The cytoplasm contained many discrete large macromolecular complexes, which were presumably mostly ribosomes. The cytoplasms of three non-dividing cells (D, E, and F in Figure 4) were searched by cross-correlation for ribosome-like particles using a low-pass-filtered ribosome model as template. Visual inspection of the search results using different thresholds showed the search had located large protein complexes within the cytoplasm, but as expected, there was no clear cutoff in the distribution of cross-correlation coefficients that could distinguish between ribosomes and other large particles (Figure 13A). Moreover, because the peak-search algorithm excluded adjacent peaks closer together than a ribosome diameter, clustered complexes were not accurately parsed. Nevertheless because a threshold of the top 500 picks clearly excluded many large protein complexes and a threshold of 2000 clearly included many smaller complexes (Figure 13B), we conclude that there were very roughly about 1250 ribosomes per cell. In comparison, exponentially growing *Saccharomyces cerevisiae* cells have ∼200,000 ribosomes each [47]. Since *S. cerevisiae* cells have up to 150 times more cytoplasm than *O. tauri* [48], [49], the concentration of ribosomes in these cells is approximately the same. In *O. tauri*, the ribosomes appeared to be uniformly distributed within the cytoplasm (Figure 13C).

### Plasma Membrane Extensions and Surface Proteins

**Figure 14:**
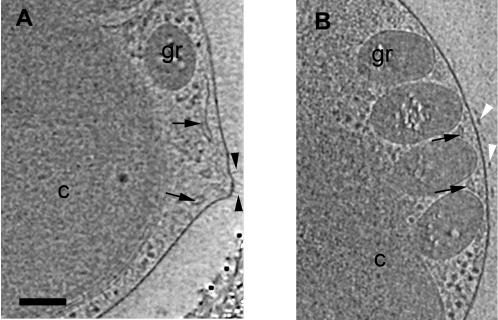
External macromolecular complexes. (A) 12-nm thick slice showing multiple filaments (black arrowheads) emerging from a cellular protuberance. Black arrows point to ER. (B) 12-nm thick slice showing macromolecular complexes (white arrowheads) on the outer surface of the plasma membrane. Scale bar 100 nm.

Three cells (6%) exhibited a bulge containing multiple 5-nm diameter filaments (Figure 14A) near sheets of ER. Two other much longer plasma membrane extensions were observed at opposite poles of an unusual cell (Figure 2E–F). One extension was at least 585 nm long (continued to the edge of the reconstructed area) and contained two internal filaments while the other was much shorter and contained a “Y” shaped inner vesicle protruding into the extension. Large macromolecular complexes were also seen decorating the outer surface of cells (Figure 14B). While these various specializations likely mediate contacts with other cells or substrates, their functions and mechanisms are of course at this point unclear.

### Conclusion

Despite application of all the best imaging technologies available, many fundamentally important aspects of eukaryotic ultrastructure remain elusive (including for instance, as a single example, the higher-order structure of chromatin). This study demonstrated that the emerging technique ECT can reveal the detailed ultrastructure of at least the smallest known eukaryotic cell, *O. tauri*, in an intact and near-native state. While ECT has already been applied to purified mitochondria and nuclei [7], [27], here they were seen in their cellular context along with structures that cannot be isolated (like the Golgi). Because *O. tauri* is becoming an increasingly well-developed model system as a simple plant and “minimal” eukaryotic cell, systematic high-resolution ultrastructural analysis by ECT as done here (but also of cells stalled in specific states and interesting mutants) will likely provide key insights into the organization of cells and the molecular mechanisms responsible. As examples, here we have seen that in G_1_, the nucleus and chloroplast sit opposite one another, and this may simply be because they are the largest bodies within the size-minimizing cell. While the mitochondrion seems to position itself randomly, the other major organelles appear to be organized by their relationship to the NE. The chloroplast exhibited its own division plane, and thus must possess its own segregation machinery for its internal features.

Nevertheless the resolution was limited in both space and time. As one benchmark example, while microtubules were easily resolved here, actin filaments were not, though that has been possible in other thinner specimens [8], [50]–[54]. Thus it remained unclear, for instance, what the molecular mechanisms were that established the division plane or segregated the different copies of each organelle. While future technological advances in both instrumentation and specimen preparation can be expected to improve the situation soon [6], it is unclear now which of *O. tauri*'s eukaryotic secrets will be the most interesting! Our original hope of visualizing undisturbed chromatin and mitosis in molecular detail, for instance, has been replaced by intrigue over the possibility that *O. tauri* may have remarkable and unique adaptations, since the nuclear envelope was wide open throughout most of the cell cycle and neither condensed chromosomes nor a mitotic spindle were ever seen. While it is possible that these expected features exist so briefly that we simply missed them in our sampling of 52 frozen cells, it is also possible that *O. tauri* will reveal significantly simplified mitotic mechanisms.

## Materials and Methods

### Cell growth

*Ostreococcus tauri* strain OTH95 (RCC 745 from the Roscoff Culture Collection) was grown in f/2 medium [55] made with Sigma Sea Salt to 36% salinity. Cells were grown at a constant 20°C with gentle, 60-RPM agitation on a rotary shaker in a cycle of twelve hours of light and twelve hours of dark.

### Traditional Fixation

*O. tauri* cells were harvested during early and mid exponential growth phase, and concentrated by 5,000 RCF centrifugation. The cell pellet was then mixed with a 1% glutaraldehyde made up in filtered ocean water. The cells were re-centrifuged, washed twice with cacodylate buffer, and fixed in 1% OsO4 for 30 minutes. The cells were re-washed twice with cacodylate buffer, washed with 30% ethanol for 10 minutes, and then mixed with 30% ethanol and 4% uranyl acetate for 20 minutes. Finally cells were then successively dehydrated with a series of ethanol washes and embedded in Epon.

### High-pressure fixation and freeze substitution

Cells were harvested by centrifugation as above. The cell pellet was high pressure-frozen in a BAL-TEC HPM 010 freezer (BAL-TEC, Liechtenstein). The samples were freeze substituted with 1% osmium tetroxide and 0.1% uranyl acetate in acetone using an EM AFS (Leica, Vienna). Samples were held at −90°C for 18 hours, warmed to −25°C at a rate of 5°C/hr, held at −25°C for 12 hours, and then warmed to room temperature at a rate of 5°C/hr. The samples were then rinsed in pure acetone, infiltrated and embedded in Epon-Araldite resin.

### Plunge Freezing

Cells were harvested by centrifugation as above, then resuspended, and combined with 10 nm gold fiducial markers. This sample was applied to glow-discharged R 2/2 Quantifoil grids (SPI Supplies), excess media was removed by a single 1 s blot (−4 mm offset, 1 s drain time), and vitrified by plunging into liquid ethane in a Vitrobot (FEI) [56].

### Electron cryotomography

Plunge-frozen grids were loaded into “flip-flop” tilt rotation holders [19] and imaged in a 300 keV, FEG, G2 Polara transmission electron microscope (FEI) equipped with an energy filter (Gatan). Throughout loading and imaging, samples were cooled with liquid nitrogen [57]. Sequentially tilted, energy-filtered (slit width 20 eV) images of individual cells were collected automatically using either the UCSF tomography [58] or Leginon software packages [59] in 2° increments up to +/−∼56–60° along either one (36 cells) or two (16 cells) axes [19] at 18,000× magnification, resulting in a CCD pixel size of 1.2 nm. The cumulative dose for each tilt series was 165 e^−^/Å^2^.

### Image processing

Images were aligned using the gold fiducials and three-dimensional reconstructions were calculated and visualized using the IMOD software package [60]. Reconstructions were denoised using thirty cycles of non-linear anisotropic diffusion with parameter lambda = 0.3 [61] using Bsoft [62] and the Peach distributed-computing system [63]. Denoised 3-D reconstructions were manually segmented using the Amira software package (Mercury Computer System, Inc.) on a Wacom Cintiq 21UX display. Volume and surface area estimates of the segmented volumes were also made using Amira. Cross-correlation searches were done using the MolMatch software [64] and an appropriately scaled and low pass-filtered cryo-EM reconstruction of a ribosome [65] as template. Cytoplasmic peaks were ranked based upon cross-correlation coefficients and then displayed using Amira.

## Supporting Information

Video S1 Electron cryotomographic reconstruction of an *Ostreococcus tauri* cell. The three-dimensional reconstruction of a single cell is shown slice-by-slice, from which the segmented model emerges in a second pass. After the various subcellular organelles are identified, the substructure of first the Golgi body and then the mitochondrion are shown in isolation, including the position and shape of the cristae junctions.
(5.06 MB MOV)
